# Plasma D-dimer and prothrombin fragment 1+2 in evaluating the occurrence of venous thromboembolism in advanced cancer patients and the effect of preventive anticoagulant therapy

**DOI:** 10.5937/jomb0-55897

**Published:** 2025-09-05

**Authors:** Yongliang Huang, Yongchun Xu, Chuanqing Ke, Baiping Zhang, Ying Zhong, Huiying Fu

**Affiliations:** 1 The 908th Hospital of Chinese People's Liberation Army Joint Logistic Support Force, Department of Gastroenterology, Nanchang, Jiangxi Province, China; 2 The 908th Hospital of Chinese People's Liberation Army Joint Logistic Support Force, Department of Oncology, Nanchang, Jiangxi Province, China; 3 The 908th Hospital of Chinese People's Liberation Army Joint Logistic Support Force, Department of Clinic, Nanchang, Jiangxi Province, China; 4 The 908th Hospital of Chinese People's Liberation Army Joint Logistic Support Force, Department of Central Sterile Supply, Nanchang, Jiangxi Province, China

**Keywords:** D-dimer (DD), prothrombin fragment 1+2 (PF1+2), advanced cancer, venous thromboembolism (VTE), risk assessment, D-dimer (DD), protrombinski fragment 1+2 (PF1+2), uznapredovali karcinom, venska tromboembolija (VTE), procena rizika

## Abstract

**Background:**

This study aimed to evaluate the role of D-dimer (DD) and prothrombin fragment 1+2 (PF1+2) in assessing the risk of venous thromboembolism (VTE) in advanced cancer patients, as well as the impact of preventive anticoagulant therapy.

**Methods:**

A total of 137 advanced cancer patients admitted to the 908th Hospital of the Chinese People's Liberation Army Joint Logistic Support Force Hospital between February 2023 and June 2024 were included. Patients were divided into two groups based on the presence (VTE group, n=49) or absence (non-VTE group, n=88) of VTE. Blood tests were performed at admission, and the relationship between DD, PF1+2, and VTE risk was analysed. Patients without VTE were further categorised into two treatment groups: the preventive anticoagulant treatment group (AG, n=48) and the conventional treatment group (RT, n=40), based on their preference. The incidence of VTE in both groups was compared to assess the effectiveness of preventive anticoagulant therapy.

**Results:**

Before chemotherapy, DD levels were significantly higher in patients who developed VTE than those who did not. Both DD and PF1+2 were found to be independent risk factors for VTE after chemotherapy. The incidence of VTE was lower in the AG group than in the RT group, with a statistically significant difference.

**Conclusions:**

DD and PF1+2 are reliable indicators for assessing VTE risk in advanced cancer patients and can help guide the use of preventive anticoagulant therapy.

## Abstract (sr)

**Uvod:**

Ova studija imala je za cilj da proceni ulogu D-dimera (DD) i protrombinskog fragmenta 1+2 (PF1+2) u proceni rizika od venske tromboembolije (VTE) kod pacijenata sa uznapredovalim karcinomom, kao i uticaj preventivne antikoagulantne terapije.

**Metode:**

U studiju je uključeno ukupno 137 pacijenata sa uznapredovalim karcinomom, hospitalizovanih u 908. bolnici Združene logističke podrške Narodnooslobodilačke vojske Kine između februara 2023. i juna 2024. Pacijenti su podeljeni u dve grupe na osnovu prisustva (VTE grupa, n=49) ili odsustva (grupa bez VTE, n=88) VTE. Krvne analize su sprovedene pri prijemu, a analizirana je povezanost između DD, PF1+2 i rizika od VTE. Pacijenti bez VTE su dodatno raspoređeni u dve grupe lečenja: grupu sa preventivnom antikoagulantnom terapijom (AG, n=48) i grupu sa standardnim lečenjem (RT , n=40), na osnovu njihovog izbora. Upoređena je incidencija VTE u obe grupe kako bi se procenila efikasnost preventivne antikoagulantne terapije.

**Rezultati:**

Pre hemoterapije, nivo DD bio je značajno viši kod pacijenata kod kojih se razvio VTE u poređenju sa onima kod kojih nije. Nakon hemoterapije, i DD i PF1+2 pokazali su se kao nezavisni faktori rizika za VTE. Incidencija VTE bila je niža u AG grupi u odnosu na RT grupu, sa statistički značajnom razlikom.

**Zaključak:**

DD i PF1+2 su pouzdani pokazatelji za procenu rizika od VTE kod pacijenata sa uznapredovalim karcinomom i mogu pomoći u usmeravanju primene preventivne antikoagulantne terapije.

## Introduction

Patients with advanced cancer face an increased risk of venous thromboembolism (VTE) due to a combination of factors that disrupt normal coagulation and blood flow. These factors include the production of procoagulant substances by the tumour, tumour invasion of the vascular walls, impaired blood flow due to tumour burden, and the effects of treatments such as chemotherapy and immobility [1] [2]. Additionally, cancer-associated hypercoagulability, often referred to as “Trousseau’s syndrome,” further heightens the likelihood of thrombus formation [3] [4] [5]. VTE, which encompasses both deep vein thrombosis (DVT) and pulmonary embolism (PE), poses a significant threat to the prognosis and quality of life of cancer patients. DVT, typically presenting as a clot in the deep veins of the legs, can cause pain, swelling, and redness in the affected limbs. If the clot detaches and travels through the bloodstream, it can result in PE, which is a life-threatening complication that causes symptoms such as chest pain, shortness of breath, hemoptysis, and even death [6] [7] [8].

Given the high risk of VTE in advanced cancer patients, timely diagnosis and prevention are critical. A key challenge in clinical practice is identifying patients at the highest risk of VTE so that appropriate preventive measures can be implemented. Traditionally, preventive strategies include using anticoagulant therapy, mobilisation strategies, and sometimes inferior vena cava filters [9]. However, identifying the most effective method for risk assessment and therapeutic intervention remains an active research area.

D-dimer (DD) and prothrombin fragment 1+2 (PF1+2) are two biomarkers increasingly used to evaluate VTE risk in cancer patients. DD is a fibrin degradation product released when fibrin clots are broken down, and elevated levels are often associated with thrombotic activity in the body. DD has long been used as a diagnostic tool for excluding DVT and PE, but elevated levels are not always specific to thrombosis, as they can also be seen in conditions such as infections, inflammation, and tumours [10] [11]. On the other hand, PF1+2 is a prothrombin fragment generated during coagulation, and its presence at elevated levels reflects the activation of the coagulation cascade. Increased PF1+2 levels have been linked to higher risks of thromboembolic events, and it has been proposed as a helpful marker in assessing coagulation status in cancer patients [12] [13].

While both DD and PF1+2 have demonstrated potential in assessing the risk of VTE, these markers are not used in isolation. They should be considered alongside other clinical indicators and diagnostic tools. Furthermore, despite the widespread use of anticoagulation therapy to prevent VTE in cancer patients, the impact of prophylactic anticoagulant therapy can vary based on factors such as tumour type, chemotherapy regimen, and patient comorbidities. Given the complexity of cancer patients’ clinical profiles, it remains unclear how best to tailor anticoagulation strategies, particularly when considering the potential risks of bleeding complications that accompany anticoagulant therapy [14] [15].

The primary goal of this study is to explore the role of DD and PF1+2 as reliable indicators for evaluating the risk of VTE in advanced cancer patients, with a particular focus on their utility in guiding preventive anticoagulant therapy. This study will assess whether these biomarkers can help predict the occurrence of VTE before and after chemotherapy and whether they can assist in determining which patients are most likely to benefit from prophylactic anticoagulant therapy. Another key aspect of this research is to investigate how different chemotherapy regimens and cancer types influence VTE risk and the efficacy of anticoagulant interventions.

The novelty of this study lies in its approach to evaluating both DD and PF1+2 as dual biomarkers in predicting VTE risk in advanced cancer patients. Moreover, the study not only investigates their potential as diagnostic tools but also aims to assess their role in guiding the implementation of preventive anticoagulant therapy. The study provides new insights into the effectiveness of tailored anticoagulation strategies by comparing the outcomes of different anticoagulant regimens, including low-molecular-weight heparin. This approach could lead to more personalised treatment regimens, improving patient outcomes while minimising the risk of bleeding complications. Additionally, by exploring the interaction between these biomarkers and different chemotherapy protocols, the study offers new data that could inform future clinical practice regarding thrombosis prevention in cancer care.

## Materials and methods

### Subjects

One hundred thirty-seven advanced cancer patients were enrolled in the 908th Hospital of the Chinese People’s Liberation Army Joint Logistic Support Force from February 2023 to June 2024. According to whether VTE occurred, the patients were divided into subjects with VTE (n=49) and subjects without VTE (n=88). Informed consent was obtained from all patients. The inclusion and exclusion criteria for patients are presented in Table 1.

**Table 1:** Inclusion criteria.

Serial number	Inclusion criteria
1	Advanced cancer patients, 18 years old or older
2	No anticoagulant therapy was given during the treatment period
3	No visible bleeding tendency
4	There are high-risk factors for VTE, such as long-term bed rest, surgery, and chemotherapy
5	Patients cooperating with trial and signing informed consent forms
Serial number	Exclusion criteria
1	Patients who have received anticoagulant therapy
2	The presence of a visible bleeding tendency
3	Presence of other serious cardiovascular or haematological diseases
4	Pregnant or lactating women
5	Patients unable to cooperate with trial requirements

### VTE ascertainment

To perform vascular scanning, patients should take off their upper clothes and put on special examination clothes provided by the hospital, and a lying position is needed so that the doctor can better perform the examination. Before starting the scan, the doctor checked the parts coated with a gel layer. A probe was adopted to scan blood vessels. The probe gently moved over the patient’s skin, and the ultrasound waves reflected the blood flow in the vessel. According to the scan results, the blood vessels’ health was assessed for further diagnosis and treatment. This vascular scan is a non-invasive diagnostic technique that helps doctors better understand the condition of blood vessels. With this kind of scanning, doctors can detect vascular problems early and take corresponding treatment measures to protect patients’ health.

### Routine blood test

During hospitalisation, venous blood samples were collected from patients before and following treatment. Fasting was required for sample collection, and then blood was collected from the median vein of the elbow on the same side. After blood collection, samples were sent to the laboratory department of the hospital for testing. The clinical laboratory tested blood samples for routine blood tests using a whole blood cell analyser. A blood routine test is a commonly adopted test method to evaluate the patient’s blood condition and health status. It includes measuring and analysing indicators such as red blood cells, white blood cells, and platelets in blood. The blood routine test shows the patient’s blood cell count, hemoglobin level, hematocrit, white blood cell differential count, and platelet count. Regular blood routine tests can monitor the blood cell status of patients and detect and deal with possible abnormalities in time. This helps doctors assess the effects of the treatment and adjust the treatment plan to provide the best treatment outcomes.

### DD testing

Plasma DD levels were measured after the 2nd, 4th, and 6th cycles of chemotherapy. To ensure accurate test results, patients should avoid eating too fat and high-protein food the day before the inspection and avoid a large number of drinks because the alcohol in the blood directly affects the test result. Blood samples were put into the automatic coagulation analyser CA-1500 and then operated according to the equipment’s instructions. The analyser would automatically perform coagulation analysis and output the results. The instructions and operating procedures of the equipment needed to be strictly followed to ensure that the samples were properly placed and that the equipment was functioning properly.

### PF1+2 testing

Enzyme-linked immunosorbent assay (ELISA) is a commonly adopted experimental technique to determine the levels of specific proteins or molecules in serum [16] [17]. This article used ELISA to measure serum PF1+2 levels. The serum sample was added to a microplate already coated with PF1+2 antibody, incubated at room temperature for a while to allow PF1+2 to bind to the antibody, and the unbound material was washed away. The horseradish peroxidase-labelled secondary antibody was then added to form the complex, and the unbound secondary antibody was washed away. The substrate was added and reacted with the enzyme-labelled secondary antibody to produce a measurable signal. Finally, the optical density of the substrate reaction product was measured using a microplate reader to determine the level of PF1+2.

### Preventive anticoagulant therapy intervention effect

The VTE group was divided into the preventive anticoagulant therapy group (AG, n=48) and normal group (RT, n=40), the AG group using low molecular heparin anticoagulation, and the RT group using routine treatment. Fourteen days as a course of treatment, at the first, second, and third courses after treatment, the probability of VTE in the two groups was counted to verify the effect of a preventive anticoagulant intervention.

### Statistical analysis

SPSS 26.0 software was adopted, and the measurement data were presented as mean ± sd. Two independent sample t-tests compared the distinctions, the multi-point contrast within the group was analysed by variance analysis, the LSD-t test was adopted to contrast two time points, and Pearson correlation analysis was adopted for the correlation between indicators. Multivariate Logistic analysis was adopted to analyse the risk factors of VTE in advanced cancer patients.

## Results

### General data of patients

There were no significant differences between the two groups regarding gender, age, tumour metastasis, or other demographic characteristics (P>0.05), suggesting that the groups were comparable for analysis. This implies that these factors did not confound the results, allowing for a valid comparison between the groups. The general characteristics of the patient groups are summarised in Table 2.

**Table 2:** Contrast of general conditions of patients.

Grouping	Gender (male/female)	Age (years old)	Whether to transfer (yes/no)
VTE group	23/26	63.5±11.45	21/28
Non-VTE group	42/46	63.4±10.72	36/52

### The relationship between blood routine and VTE

**Figure 1:**
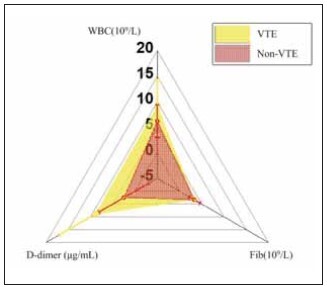
Contrast of subjects’ blood routine tests.

A comparison of the blood routine results between the two groups revealed significant differences in the white blood cell count and D-dimer (DD) levels before treatment (P<0.05), suggesting a potential association between these markers and VTE. However, no significant differences were observed for platelet count and fibrinogen levels, indicating that these factors may not be directly related to the occurrence of VTE in this cohort. Figure 1 illustrates the blood routine test results for both groups.

### Change in plasma DD

**Figure 2:**
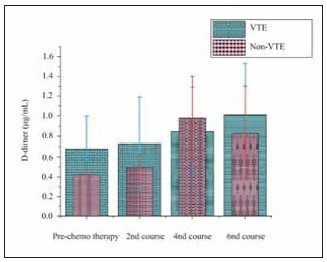
Changes of plasma DD in subjects.

Before chemotherapy, plasma DD levels were higher than the critical threshold in all subjects, with those in the VTE group exhibiting notably elevated DD levels compared to those in the non-VTE group. During chemotherapy, plasma DD levels increased further, particularly in patients who developed VTE. Although the DD levels in patients with VTE rose during later stages of treatment, the increase was not statistically significant when compared to the non-VTE group, possibly due to the confounding effect of anticoagulant therapy (which was administered to some patients). Figure 2 demonstrates the temporal changes in plasma DD levels throughout chemotherapy.

### Change of PF1+2

**Figure 3:**
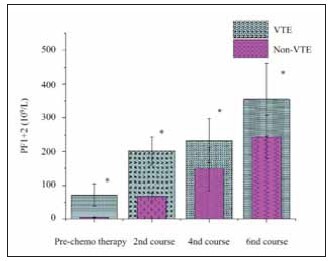
PF1+2 changes in subjects during remedy.<br>Note: * represents a visible distinction

Levels of prothrombin fragment 1+2 (PF1+2) increased progressively throughout chemotherapy for all patients. Importantly, patients who developed VTE exhibited significantly higher PF1+2 levels than those in the non-VTE group (P<0.05). These findings suggest that PF1+2 could be a useful marker for identifying patients at higher risk for VTE. The changes in PF1+2 levels during chemotherapy are shown in Figure 3.

### Logistic analysis of DD, PF1+2, and VTE

**Figure 4:**
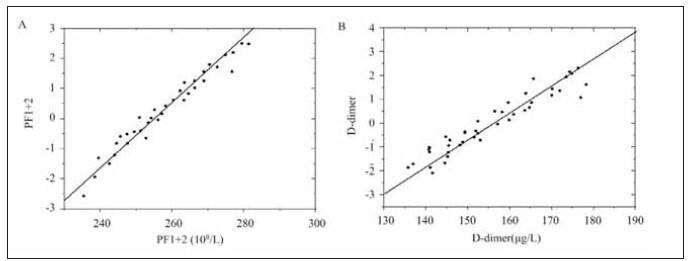
Logistic analysis contrast of DD, PF1+2, and VTE.<br>A: Logistic analysis of PF1+2; B: Logistic analysis of DD

Logistic regression analysis revealed that elevated plasma DD and PF1+2 levels after chemotherapy were independent risk factors for VTE. Specifically, patients with higher levels of these biomarkers were more likely to develop VTE during their treatment course. The results of the logistic analysis are presented in Figure 4, indicating the odds ratios for DD and PF1+2 in predicting VTE risk.

### Effect of preventive anticoagulant therapy intervention

**Figure 5:**
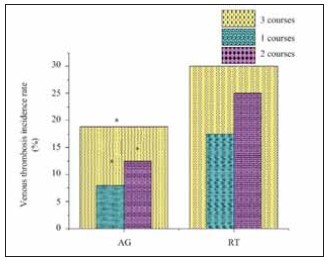
The probability of VTE occurring in subjects adopting AG and RT during the first, second, and third course of treatment.<br>Note: * represents PF1+2 had visible distinction

The impact of preventive anticoagulant therapy on VTE incidence was assessed during three courses of chemotherapy. In the first course, 4 subjects receiving anticoagulant therapy (AG) developed VTE, while 7 subjects in the radiotherapy (RT) group developed VTE. During the second course, 6 subjects on AG and 10 on RT developed VTE. Notably, by the third course, the incidence of VTE was significantly higher in the RT group, with 94 cases of VTE occurring among subjects receiving RT, compared to just 12 cases in the AG group. The statistical analysis confirmed that the difference in VTE incidence between AG and RT groups was significant across all treatment courses (P<0.05). These results are presented in Figure 5, which shows the changing incidence of VTE across the three courses of chemotherapy.

## Discussion

Studies have found that there may be various mechanisms leading to VTE in cancer patients [18] [19] [20] [21]. First, tumours can create vortexes in blood vessels by compressing and narrowing the lumen, increasing the risk of thrombosis. Secondly, the blood viscosity of tumour patients is increased, and the blood flow velocity is slowed down, which is also prone to thrombosis. In addition, some drugs and intravenous chemotherapy may stimulate peripheral blood vessels, leading to vascular damage, which is one of the basic factors of thrombosis. Viral or bacterial infections can also prompt the formation of blood clots in the microcirculation. In addition, tumour debris or secreted products and coagulation-activating substances such as bacteremia can also cause thrombosis after entering the blood [22]. In this article, DD and PF1+2 were evaluated as indicators to evaluate VTE in advanced cancer patients, and the intervention effect of prophylactic anticoagulation on VTE was explored. It is found that the outcome of DD and PF1+2 in evaluating the occurrence of VTE in advanced cancer patients is visible. DD is a blood marker produced by the degradation of blood clots, and its level can reflect the degree of thrombosis and activation of the fibrinolytic system [23] [24]. PF1+2 is a prothrombin fragment, and its level can indirectly reflect the activation of the coagulation system [25] [26].

Detecting these two markers can help doctors evaluate the risk of thrombosis and coagulation function of patients, which is of great significance for diagnosing and remedying thrombotic diseases. Studies have shown that elevated levels of DD and PF1+2 are associated with an increased risk of VTE. Therefore, evaluating DD and PF1+2 levels can help doctors detect the risk of thrombosis in patients in time and take corresponding preventive measures. It is essential for patients with advanced tumours because they are, due to the tumour and remedy, more likely to have thromboembolism, and the prognosis is worse. Secondly, intervention studies of preventive anticoagulation therapy have been conducted. The results showed that prophylactic anticoagulant therapy can visibly reduce the incidence of VTE in advanced cancer patients. This indicates that prophylactic anticoagulation therapy is meaningful for reducing the risk of VTE in advanced cancer patients. However, it has also been noted that prophylactic anticoagulant therapy may increase the risk of bleeding. Therefore, deciding whether to undergo preventive anticoagulation therapy is necessary to consider the patient’s VTE risk and bleeding risk comprehensively.

In advanced cancer patients before chemotherapy, the plasma DD level was higher than the critical value, and the DD level was higher in subjects with VTE as against subjects without VTE. However, in the process of chemotherapy, plasma DD in subjects with VTE had increased in the later remedy, but it had no visible distinctions as against subjects without VTE. This may be due to the effect of anticoagulant therapy. In general, the plasma DD level of subjects gradually increased with the progress of chemotherapy. These findings suggest that DD levels are closely associated with VTE in advanced cancer patients. VTE is a common complication that has a major impact on a patient’s quality of life and prognosis. Therefore, monitoring DD levels may help to detect the risk of VTE early and take preventive measures accordingly. In addition, anticoagulant therapy may play a role in controlling DD levels. Although the level of DD increased in the subjects with VTE, it was not visibly different from those without VTE. This may be due to the effectiveness of anticoagulant therapy, which can reduce thrombosis and alleviate inflammatory reactions. However, with the progress of chemotherapy, the plasma DD of subjects gradually increased. This may be due to the tumour’s increased growth and metabolic activity. In addition, chemotherapy drugs may affect blood coagulation function, further promoting DD increase. Prophylactic anticoagulation may be necessary for high-risk patients, but anticoagulationmay need to be evaluated more carefully for low-risk patients. In addition, it has been found that the effect of prophylactic anticoagulation may be influenced by tumour type and remedy regimen. Different tumour types and remedy regimens may lead to varying degrees of coagulation abnormalities and VTE risk. Therefore, it is necessary to consider patients’ individual conditions and remedy options when developing preventive anticoagulant therapy strategies.

In conclusion, DD and PF1+2 have important clinical value as indicators for evaluating the occurrence of VTE in advanced cancer patients. Prophylactic anticoagulation therapy can visibly reduce the incidence of VTE in advanced cancer patients, but the risk of VTE and bleeding should be carefully weighed. Further studies will be helpful in optimising prophylaxis [27] [28].

Ay and colleagues found that elevated levels of DD and PF1+2 were significant predictors of VTE in cancer patients, highlighting their potential as biomarkers for assessing VTE risk in this population [29]. Their study, which followed 821 patients over a median of 501 days, showed that patients with elevated levels of these markers had a significantly higher risk of developing VTE, particularly when both DD and PF1+2 levels were elevated. Our study supports these findings by demonstrating that DD and PF1+2 are also independent risk factors for VTE in advanced cancer patients, with higher levels observed in the VTE group before chemotherapy. Furthermore, our research extends this by investigating the impact of preventive anticoagulant therapy, showing that patients who received preventive anticoagulants (AG group) had a lower incidence of VTE compared to those in the conventional anticoagulant treatment group (RT group), suggesting that DD and PF1+2 could also guide therapeutic decision-making. Both studies underline the importance of these biomarkers in identifying high-risk patients and potentially improving outcomes through early intervention.

Chen and colleagues [30] explored the prognostic significance of an elevated plasma D-dimer cut-off value in advanced non-small cell lung cancer (NSCLC), suggesting that a higher threshold of 981 ng/mL better predicts overall survival than the commonly used 500 ng/mL cut-off. Their study, which analysed 233 patients, found that patients with D-dimer levels above 981 ng/mL had significantly worse survival outcomes than those with lower levels, indicating that this marker can serve as a more accurate prognostic tool for advanced NSCLC. Our study further supports the utility of D-dimer in cancer-related thrombosis by demonstrating that D-dimer, along with PF1+2, can predict the risk of venous thromboembolism (VTE) in advanced cancer patients. While Chen et al. focused on the prognostic value for survival, our research highlights the role of D-dimer and PF1+2 in prognosis and in guiding the management of VTE through preventive anticoagulant therapy. Both studies emphasise the importance of refining D-dimer thresholds to improve patient outcomes, whether predicting survival or preventing thromboembolic events.

As against other experiments [27] [28], DD and PF1+2 assessment can help physicians detect possible VTE risk in advanced cancer patients in time, perform preventive anticoagulation intervention in advance, and reduce the occurrence of complications. This assessment method can more accurately assess the risk of thrombosis in patients and help to develop personalised remedy plans. However, the evaluation results of DD and PF1+2 may be affected by other factors, such as age, gender, and inflammatory state, and there may be a certain misdiagnosis rate. In addition, prophylactic anticoagulant therapy may increase the risk of bleeding, which requires doctors to consider and avoid unnecessary remedies carefully. Meanwhile, DD and PF1+2 levels evaluation require special laboratory tests, which may increase medical and time costs. In conclusion, although DD and PF1+2 assessment plays a major role in helping identify patients at risk of thrombosis, physicians must carefully consider various factors when deciding whether to perform prophylactic anticoagulation intervention. They should be aware of the limitations of the assessment results and the possible additional costs in practice.

One limitation of our study is the potential impact of confounding factors, such as age, gender, and inflammation, which may affect the accuracy of DD and PF1+2 in predicting VTE risk. Additionally, there may be misdiagnosis risks, and using specialised tests could increase costs and time. Prophylactic anticoagulation therapy, while effective in reducing VTE, may raise bleeding risks, requiring careful risk assessment. Tumour type and treatment regimens may also influence DD and PF1+2 levels, complicating the generalisation of our findings.

## Conclusion

DD and PF1+2 are reliable markers for assessing VTE risk in advanced cancer patients. However, when considering prophylactic anticoagulation, the risk of bleeding must be carefully weighed. Decisions should be individualised based on the patient’s condition, tumour type, and treatment options. While high-risk patients may benefit from anticoagulation, low-risk patients require a more cautious approach to balance risks and benefits.

## Dodatak

### Conflict of interest statement

All the authors declare that they have no conflict of interest in this work.

## References

[1] Momenzadeh K, Yeritsyan D, Mortensen S, Kheir N, Khak M, Caro D, et al (2024). While the Incidence of Venous Thromboembolism After Shoulder Arthroscopy Is Low, the Risk Factors Are a Body Mass Index Greater than 30 and Hypertension. Arthroscopy, Sports Medicine, and Rehabilitation.

[2] Elkbuli A, Patel H, Breeding T, Nasef H, Chin B, Wright D D, et al (2024). Racial Distribution and Associated Outcomes for Patients With and Without Severe-Isolated Traumatic Brain Injuries Following Venous Thromboembolism Prophylaxis. Am Surg.

[3] Yang S, Zhang Y, Jiao X, Liu J, Wang W, Kuang T, et al (2023). Padua prediction score may be inappropriate for VTE risk assessment in hospitalized patients with acute respiratory conditions: A Chinese single-center cohort study. Int J Cardiol Heart Vasc.

[4] Gu S, Chen Y E, Lei M, Li J, Li W, Zhang M, et al (2023). Effect of different application duration of a venous foot pump on prevention of venous thromboembolism after hip and knee arthroplasty: a multicenter prospective clinical trial. BMC Musculoskelet Disord.

[5] Kim J, Jeong W K, Kim J M, Ha S Y, Kim K (2024). Refining MRI-based criteria for portal vein invasion in hepatocellular carcinoma: improving sensitivity beyond portal vein tumor thrombosis. Abdom Radiol (NY).

[6] Rajendram R, Hussain A, Mahmood N, Kharal M (2023). Correction: Feasibility of using a handheld ultrasound device to detect and characterize shunt and deep vein thrombosis in patients with COVID-19: an observational study. The Ultrasound Journal.

[7] Jones D H, Lin B, Brabham D, Trinidad B (2023). Removal of an infected pulmonary artery fibroelastoma disguised as a presentation of pulmonary embolism using a percutaneous suction thrombectomy device. J Vasc Surg Cases Innov Tech.

[8] Nze Ossima A, Ngaleu Siaha B F, Mimouni M, Mezaour N, Darlington M, Berard L, et al (2023). Cost-effectiveness of modified diagnostic strategy to safely rule-out pulmonary embolism in the emergency department: a non-inferiority cluster crossover randomized trial (MODIGLIA-NI). BMC Emerg Med.

[9] Chen Q, Luo J, Yang X, Chen W, Liu W, Song Z (2024). Biomarkers of Autologous Whole Blood Injection Efficacy in Patients with Chronic Spontaneous Urticaria with Autoreactivity: A Preliminary Study. Int Arch Allergy Immunol.

[10] Ozsay O, Aydin M C, Karabulut K, Basoglu M, Dilek O N (2024). Venous reconstruction thrombosis after pancreaticoduodenectomy with superior mesenteric/portal vein resection due to pancreatic cancer: an 8 years single institution experience. Acta Chir Belg.

[11] Đordević A, Grahovac B, Šegulja S, Bilić Zulle L, Roganović J (2023). Inherited Thrombophilia and Risk of Thrombosis in Children with Cancer: a Single-center Experience. Acta Med Acad.

[12] Paramitha M S, Esa D F, Hustrini N M, Mulansari N A, Hasan I, Harahap A S (2024). Secondary Polycythemia and Non-Islet Cell Tumor-induced Hypoglycemia in Advanced Hepatocellular Carcinoma: A Case Report. Acta Med Indones.

[13] Zeitouni M, Giczewska A, Lopes R D, Wojdyla D M, Christersson C, Siegbahn A, et al (2020). Clinical and Pharmacological Effects of Apixaban Dose Adjustment in the Aristotle Trial. J Am Coll Cardiol.

[14] Min L, Bao H, Bu F, Li X, Guo Q, Liu M, et al (2023). Machine-Learning-Assisted Procoagulant Extracellular Vesicle Barcode Assay toward High-Performance Evaluation of Thrombosis-Induced Death Risk in Cancer Patients. ACS Nano.

[15] Feng L, Xing H, Zhang K (2022). The therapeutic potential of traditional Chinese medicine in depression: Targeting adult hippocampal neurogenesis. Phytomedicine.

[16] Peng Y, Peng H, Ke W (2023). Influence mechanism of osteopontin on renal injury in patients with hereditary hypercalcemia by enzyme-linked immunosorbent assay. Cell Mol Biol (Noisy-le-grand).

[17] Lu Y, Wu Q, Wang L, Ji L (2024). Serological evaluation of recombinant protein antigen Tp0608 for the diagnosis of syphilis. Diagn Microbiol Infect Dis.

[18] Bhutiani N, Quinn S A, Mercer M K, Hong Y K, Stevenson M, Egger M E, et al (2019). Identifying risk factor for development of perioperative venous thromboembolism in patients with gastrointestinal malignancy. Am J Surg.

[19] Negash M, Wondmagegn T, Geremew D (2018). Comparison of RPR and ELISA with TPHA for the Diagnosis of Syphilis: Implication for Updating Syphilis Point-of-Care Tests in Ethiopia. J Immunol Res.

[20] Goldberg I, Spectre G, Raanani P, Cate H T, Leader A (2024). Clinical Challenges in Treating Cancer-Associated Thrombosis: A Clinically Oriented Review. Acta Haematol.

[21] Sugimoto T, Inoue A, Komori T, Nishizawa Y, Kagawa Y, Komatsu H, et al (2022). Investigation of the Short-Term Outcome of Perioperative Heparinization in Laparoscopic Surgery for Colorectal Cancer. Gan To Kagaku Ryoho.

[22] Harino T, Noura S, Hamabe A, Ogino T, Takeyama H, Suzuki Y, et al (2022). Impact of aspirin discontinuation on thrombotic complications in laparoscopic colorectal cancer surgery. Surg Endosc.

[23] İlhan M, Alizade E, Uzunyolcu G, Gök A F K, Gunay K, Ertekin C, et al (2022). Is emergency gastrointestinal system tumour surgery safe under treatment of antitrombotics?. Ulus Travma Acil Cerrahi Derg.

[24] Oberle L, Tatagiba M, Naros G, Machetanz K (2024). Intermittend pneumatic venous thrombembolism (VTE) prophylaxis during neurosurgical procedures. Acta Neurochir (Wien).

[25] Zimmer K, Scheer M, Scheller C, Leisz S, Strauss C, Taute B M, et al (2024). Influence of postoperative D-dimer evaluation and intraoperative use of intermittent pneumatic vein compression (IPC) on detection and development of perioperative venous thromboembolism in brain tumor surgery. Acta Neurochir (Wien).

[26] Vahtera A, Szanto T, Lassila R, Valkonen M, Sivula M, Huhtala H, et al (2021). Continuous intravenous infusion of enoxaparin controls thrombin formation more than standard subcutaneous administration in critically ill patients. A sub-study of the ENOKSI thromboprophylaxis RCT. Acta Anaesthesiol Scand.

[27] Schön M, Infante J, Pinho E Melo T, Lacerda J F, Ferro J M (2024). Cerebral venous thrombosis as a first presentation of a high-risk acute myeloid leukaemia. Acta Neurol Belg.

[28] Chai X, Zhu T, Chen Z, Zhang H, Wu X (2024). Improvements and challenges in intraperitoneal laparoscopic para-aortic lymphadenectomy: The novel 'tent-pitching' antegrade approach and vascular anatomical variations in the para-aortic region. Acta Obstet Gynecol Scand.

[29] Ay C, Vormittag R, Dunkler D, Simanek R, Chiriac A L, Drach J, et al (2009). D-Dimer and Prothrombin Fragment 1 + 2 Predict Venous Thromboembolism in Patients With Cancer: Results From the Vienna Cancer and Thrombosis Study. J Clin Oncol.

[30] Chen C, Li J, Li J, Wang X, Wang X, Du N, et al (2020). Application of an elevated plasma D-dimer cut-off value improves prognosis prediction of advanced non-small cell lung cancer. Ann Transl Med.

